# Growth charts for small sample sizes using unsupervised clustering: Application to canine early growth

**DOI:** 10.1007/s11259-022-10029-2

**Published:** 2022-11-05

**Authors:** Gabriel Kocevar, Maxime Rioland, Jérémy Laxalde, Amélie Mugnier, Achraf Adib-Lesaux, Virginie Gaillard, Jonathan Bodin

**Affiliations:** 1 Seenovate, Lyon, France; 2 Royal Canin Research Center, Aimargues, France; 3 NeoCare, Université de Toulouse, ENVT, Toulouse, France

**Keywords:** Puppy, Growth curves, Unsupervised clustering, GAMLSS, Small sample size

## Abstract

Breed-specific growth curves (GCs) are needed for neonatal puppies, but breed-specific data may be insufficient. We investigated an unsupervised clustering methodology for modeling GCs by augmenting breed-specific data with data from breeds having similar growth profiles. Puppy breeds were grouped by median growth profiles (bodyweights between birth and Day 20) using hierarchical clustering on principal components. Median bodyweights for breeds in a cluster were centered to that cluster’s median and used to model cluster GCs by Generalized Additive Models for Location, Shape and Scale. These were centered back to breed growth profiles to produce cluster-scale breed GCs. The accuracy of breed-scale GCs modeled with breed-specific data only and cluster-scale breed GCs were compared when modeled from diminishing sample sizes. A complete dataset of Labrador Retriever bodyweights (birth to Day 20) was split into training (410 puppies) and test (460 puppies) datasets. Cluster-scale breed and breed-scale GCs were modelled from defined sample sizes from the training dataset. Quality criteria were the percentages of observed data in the test dataset outside the target growth centiles of simulations. Accuracy of cluster-scale breed GCs remained consistently high down to sampling sizes of three. They slightly overestimated breed variability, but centile curves were smooth and consistent with breed-scale GCs modeled from the complete Labrador Retriever dataset. At sampling sizes ≤ 20, the quality of breed-scale GCs reduced notably. In conclusion, GCs for neonatal puppies generated using a breed-cluster hybrid methodology can be more satisfactory than GCs at purely the breed level when sample sizes are small.

**Supplementary Information:**

The online version contains supplementary material available at 10.1007/s11259-022-10029-2.

## Introduction

Monitoring the growth of children is embedded in many national health services across the world (de Onis et al. 2004). By comparing the growth trajectory of an individual with standard growth centile curves plotted against age, a health-care professional can identify abnormal growth and take the appropriate steps to diagnose underlying disorders and try to correct them (Scherdel et al. 2016). The pattern of early growth of the fetus and infant is particularly important because it can be a predictor of both short-term health and health status in adulthood according to the Developmental Origins of Health and Disease (Barker and Osmond 1986; Barker et al. 1989, 1990; Barker 1990, 2007). To ensure adequate monitoring, there are international standards for optimal growth developed by the World Health Organization (WHO) (2006) as well as national ones generated from country-specific data (Natale and Rajagopalan 2014; Tinggaard et al. 2014).

Growth curves (GCs) have also been developed for non-human species, including companion animals (Hawthorne et al. 2004; Salt et al. 2017, 2020; Cave et al. 2018; Alves 2020), livestock (Schinckel et al. 2004; Berry et al. 2005; Kuhi et al. 2010; da Silva Marinho et al. 2013; Luo et al. 2015; Sogut et al. 2016), marine animals (Allen et al. 1972; Frazer and Ladner 1986; Hernandez-Llamas and Ratkowsky 2004) and plants (Elias and Causton 1976; Poorter and Garnier 1996; Díaz-Galián et al. 2019). The rationale for GCs and their potential applications in animals extends beyond flagging any abnormal growth in an individual that requires diagnostic investigations. In cats, patterns of growth have been evaluated in order to predict the development of obesity (Cave et al. 2018). In dogs, GCs have been proposed as tool to help determine the need for supplementary feeding of neonates (Alves 2020), and to inform feeding guides for different breeds (Salt et al. 2017, 2020; Alves 2020). An understanding of growth trajectories in cattle, pigs and poultry is of economic importance to guide nutritional and environmental management, breeding strategies for different strains, and predictions of the best age for slaughter (Schinckel et al. 2004; Berry et al. 2005; Kuhi et al. 2010; da Silva Marinho et al. 2013; Luo et al. 2015; Sogut et al. 2016). Growth curves have also been generated to facilitate the use of a pig model for biomedical research (Corson et al. 2008). In wild marine species, research into growth trajectories has potential implications for ecological management and fishing strategies (Hernandez-Llamas and Ratkowsky 2004).

A range of statistical methods have been used for building GCs in these different species. These can be categorized broadly according to whether age is treated as a discrete or a continuous variable, and whether the distribution of data is a priori or determined from the dataset. For example, the Center for Disease Control and Prevention in the USA has taken age as a discrete variable without a priori data distribution in a two-stage statistical procedure for weight-for-age growth charts (Kuczmarkski et al. 2002). Age was divided into intervals (age binned) and empirical centiles for weight were smoothed using a three-parameter linear model (Guo et al. 1992), in which locally robust weighted regression was followed by polynomial regression (Cleveland 1979); Z-scores were calculated by a lambda, mu, and sigma (LMS) approximation post hoc (Kuczmarkski et al. 2002). Having a hypothesis on data distribution permits the calculation of z-scores and results in a better estimation of extreme centiles (Borghi et al. 2006). However, the distribution of data is not the same at all ages and its temporal evolution may influence the outcome of the modeling. These observations led the WHO to recommend modeling methods for growth charts that employ a priori data distribution and allow this to evolve temporally (Borghi et al. 2006). The data distribution is generally characterized by mean, standard deviation (SD) and asymmetry (skewness). Additionally, the WHO has indicated that adjustment for kurtosis should be considered if needed to prevent the distortion of fitted centiles (Borghi et al. 2006).

The statistical framework used for both the WHO’s updated 2006 growth standards for children (2006) and the recent set of growth standard charts for monitoring bodyweight of dogs (Salt et al. 2017) was Generalized Additive Models for Location, Shape and Scale (GAMLSS) (Rigby and Stasinopoulos 2005). GAMLSS is a flexible semi-parametric modeling approach comprising a series of univariate distributional regression models that can be used with any assumed distribution of the response variable, allowing skewness and kurtosis to be taken into account (GAMLSS 2021). It can be implemented with a wide variety of linear and/or non-linear and/or non-parametric smoothing functions of the explanatory variables. Unlike quantile regression methods (Koenker and Bassett 1978), with GAMLSS, the quantile charts cannot intersect and there is an explicit formula to calculate quantiles.

Interest in age-to-weight growth standards in puppies reflects evidence that obesity (Leclerc et al. 2017), and orthopedic (Dämmrich 1991; Kealy et al. 1992) and endocrine (Greco 2012) disorders have been associated with the rate of growth. Much of the work on early growth of dogs has focused on the post-weaning period, including the most comprehensive study to date that generated GCs for 24 individual breeds from the age of 2 months (Salt et al. 2017). However, there are persistently high mortality rates in neonatal puppies (approximately 10–15% in large national studies Tønnessen et al. 2012; Chastant-Maillard et al. 2017)) and the first three weeks are a critical period for puppy survival (Indrebø et al. 2007; Tønnessen et al. 2012; Vassalo et al. 2015). Several studies demonstrate an association between low birth weight of puppies and risk of death (Groppetti et al. 2015; Mila et al. 2015; Mugnier et al. 2019), yet the majority of 674 breeders in a recent survey did not recognize this (Mugnier et al. 2021). Poor growth in the first 2 days is known to be a predictor of mortality (Mila et al. 2015). It is not known however whether speed of bodyweight gain in the neonate has an impact on adult underweight or overweight in the same way as demonstrated for growth between 10 weeks and 3 months of age (Salt et al. 2020). All of these factors highlight the need for GCs for neonatal and young puppies from birth to 2 months of age when recent standard GCs start (Salt et al. 2017).

A systematic approach to modeling GCs needs to be tailored for puppies in this age group. Ideally growth charts should be breed specific to account for the large heterogeneity in weight between dog breeds (from less than 1 kg to over 100 kg). There are almost 400 breeds of dogs, and for less common breeds there will only be small sample sizes available to model GCs, and GAMLSS-based growth charts cannot be accurately estimated using a small sample size.

The main purpose of this study was to propose a method for building puppy GCs that would be appropriate in the context of small sample sizes for individual breeds, and to validate this using the Labrador Retriever as an example breed. This method was founded on unsupervised clustering of dog breeds using the evolution of median bodyweight over time for each breed. The reliability of growth centile curves created from weight measurements of a cluster of breeds (cluster-scale breed GCs) was challenged by simulating such curves with diminishing sample sizes of data from the breed of interest in the cluster. We evaluated the quality of these cluster-scale GCs for the Labrador Retriever by comparing them with classical breed-scale Labrador Retriever GCs generated with the same diminishing sample sizes. The criteria for comparing quality were based on how well distribution of observed data matched the theoretical centile values of the GCs. The hypothesis for this study was that cluster-scale breed GCs would be more accurate than purely breed-specific GCs when the sample size was small.

## Material and methods

### Studied populations

Daily bodyweight measurements of pure-bred puppies (119 different breeds) from birth to 62 days were obtained retrospectively from 291 dog breeding establishments in France as described previously (Mugnier et al. 2019). Breeders completed a questionnaire voluntarily and agreed to their data being used for the purpose of this research. Animals that died within 62 days of birth were not included in this analysis. Prior to modeling, Tukey method was applied by breed and by timepoint to remove extreme values that were higher than the 75^th^ centile plus 1.5 times the interquartile distance, or lower than the 25^th^ centile minus 1.5 times the interquartile distance (Tukey 1977).

With respect to the Labrador Retriever sub-population used as example in this study, in order to maintain consistency between simulations a complete dataset was compiled that included only those Labrador Retriever puppies without missing bodyweight measures between Day 0 (birth) and Day 20.

### Modeling of GCs

Growth curves for Days 0 to 20 were modeled with the GAMLSS 5.1–2 package in R language version 3.4.4 (Stasinopoulos et al. 2017). Box-Cox Cole and Green (Cole and Green 1992), Box-Cox T (Rigby and Stasinopoulos 2006) and Box-Cox Power Exponential (BCPE) (Rigby and Stasinopoulos 2004) distributions were tested, and BCPE was selected by Generalized Akaike Information Criterion (GAIC) as the most suitable (Taniguchi and Hirukawa 2012). The four parameters (mean, SD, skewness and kurtosis) were estimated using BCPE model and parameters were smoothed using cubic spline function. Finally, the 9^th^, 25^th^, 50^th^, 75^th^, and 91^st^ growth centile curves were generated (Pollock 1993).

### Generation of cluster-scale breed GCs using an unsupervised clustering approach

**Fig. 1:**
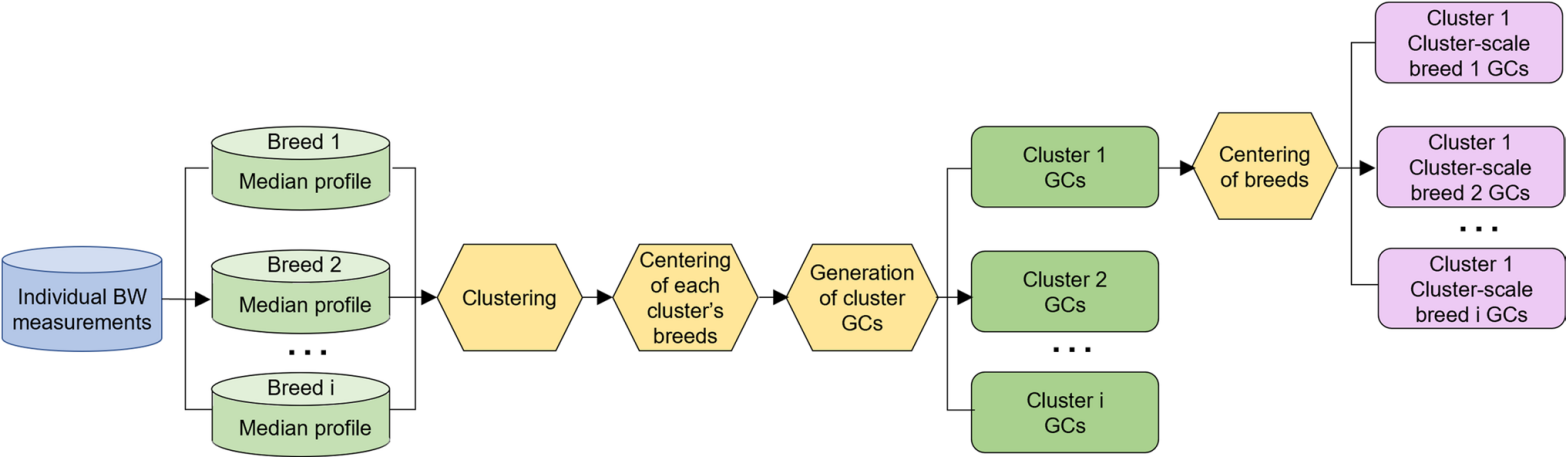
Key steps in modeling cluster-scale breed GCs using an unsupervised clustering approach. BW, bodyweight; GCs, growth curves

An overview of the methodology for generating cluster-scale breed GCs is presented in Fig. 1. In brief, there were five main steps:

1. Computation of the profile of median bodyweight evolution across time points for each available breed,
2. Unsupervised clustering using hierarchical clustering on principal components of breeds based on breed profiles of median bodyweight evolution,
3. Centering of each breed in a cluster to the median bodyweight value of that cluster at each time point,
4. Computation of cluster growth centile curves,
5. Centering of the cluster growth centile curves to the median bodyweight value of each breed at each time point to generate cluster-scale breed GCs.

The methodology aimed to maximize inter-cluster variability and minimize intra-cluster variability.

### Evaluation of the accuracy of breed-scale and cluster-scale GCs when breed sample size was small

The accuracies of both cluster-scale breed GCs and breed-scale GCs were assessed for the Labrador Retriever using modeling simulations for seven different sample sizes of Labrador Retriever puppies. For each of the 100 simulations, puppies from the complete Labrador Retriever dataset (n = 860) were randomly divided into a training dataset of 410 puppies and a test dataset of 450 puppies. The training dataset was then randomly sampled for sample sizes of 410, 200, 100, 30, 20, 10 and 3 puppies. For each sample size, breed-scale GCs and cluster-scale GCs were simulated using the modeling methodology previously described. Breed-scale GCs were simulated using only the Labrador Retriever training dataset. Cluster-scale breed growth charts were simulated using the Labrador Retriever training dataset and all data from other breeds belonging to its defined cluster. This process was repeated 100 times resulting in 700 cluster-scale curves and 100 breed-scale curves.

The quality of each simulated growth centile curve was estimated for each timepoint using the corresponding test dataset of observed bodyweights. The percentages of observed bodyweight measurements that were higher than the modeled bodyweight values for the 75^th^ or 91^st^ centiles, and lower than those for the 9^th^ and 25^th^ centiles, were compared against the centile targets (i.e. 9% for the 9^th^ and 91^st^ centiles and 25% for the 25^th^ and 75^th^ centiles). These percentages of observed bodyweight measures are referred to as the quality criteria or estimations.

### Statistical analysis

Descriptive statistics were calculated for quality estimations as appropriate, including mean, 95% confidence intervals (CIs) median, range, SD and coefficient of variation (CV) using R software (2014).

## Results

After removal of extreme values, the global dataset contained 390,202 body weight measurements from 15,887 individual puppies across 116 breeds from the five previously reported size categories (extra-small, mini, medium, maxi and giant) (Salt et al. 2017). The most represented breed in the dataset was the Labrador Retriever (n = 1,915 puppies).

### Breed-scale GCs: example of the Labrador Retriever

**Fig. 2:**
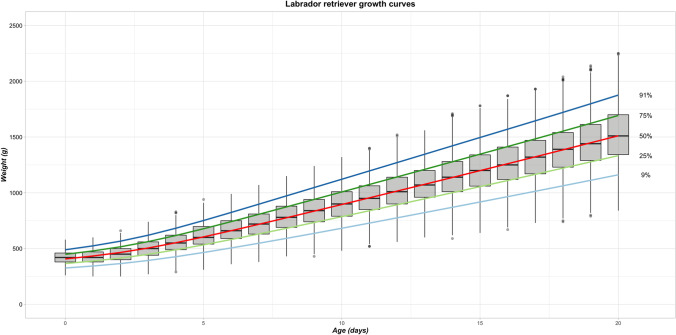
Breed-scale growth centile curves built from the complete Labrador Retriever bodyweight dataset. The chart shows growth centile curves modeled from the complete Labrador Retriever dataset (860 puppies) overlaying a box and whisker chart of the distribution of observed data per timepoint (Days 0 to 20). Boxes outline the interquartile range, the horizontal bars are the median, the top and bottom whiskers extend to largest and smallest weight measures, respectively, within 1.5 times the interquartile range, and filled circles represent data points outside these ranges. Close alignment between the box and whisker charts and centile curves demonstrates the accuracy of the modeling

**Fig. 3:**
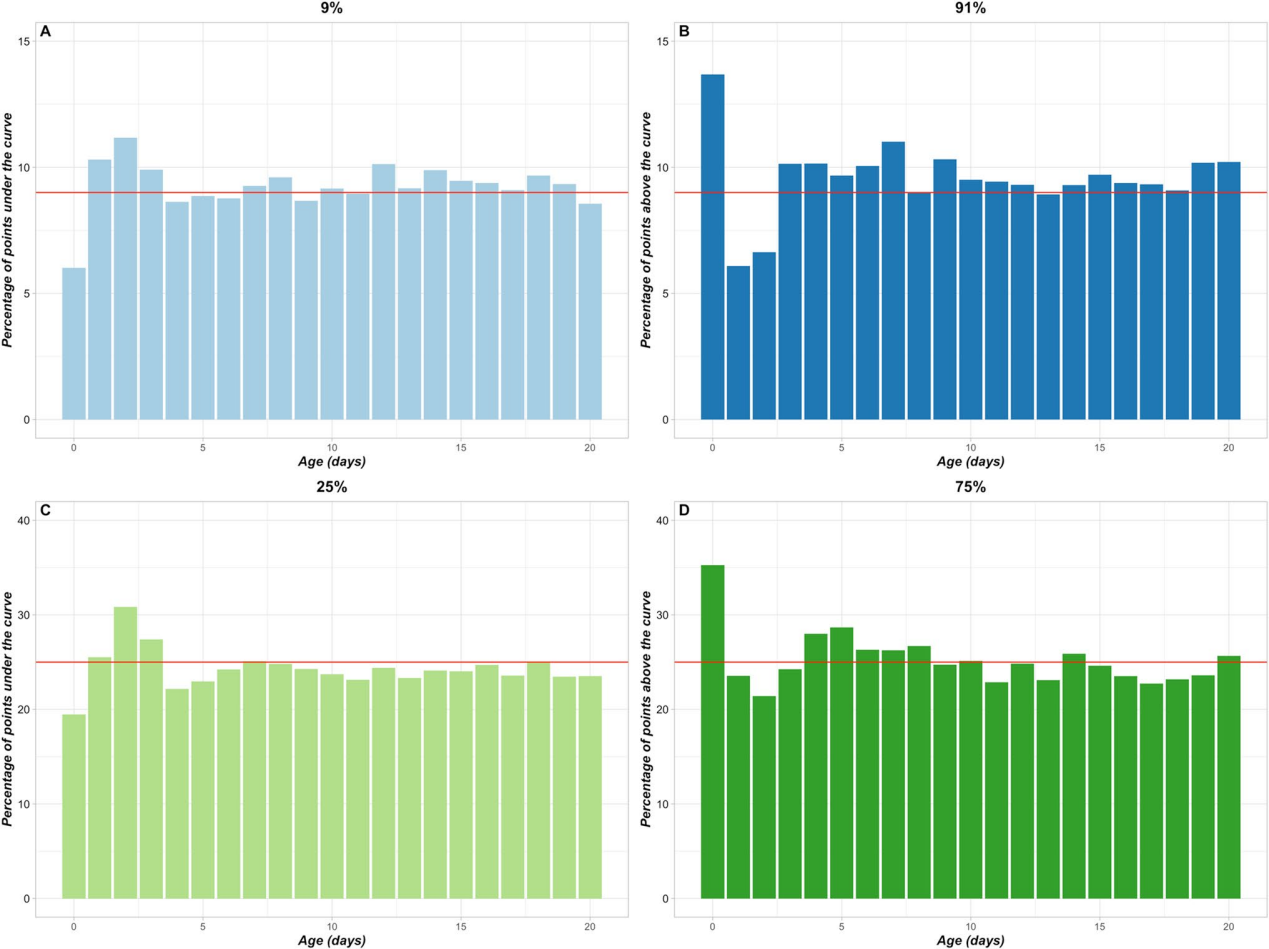
Quality estimations of breed-scale Labrador Retriever growth centile curves. Charts show the percentage of observed Labrador Retriever BW measurements by timepoint that were outside the modeled target centile for breed-scale Labrador Retriever GCs developed from the complete Labrador Retriever dataset (860 puppies). The red line indicates the target percentage. The bars show the percentages of observed BWs that were lower than the BW value of the 9^th^ centile (**A**), higher than the BW value of 91^st^ centile (**B**), lower than the BW value of the 25^th^ centile (**C**), and higher than the BW value of the 75^th^ centile (**D**). Quality criteria were close to their targets for all centiles. BW, bodyweight; GCs, growth curves

Breed-scale growth centile curves for the Labrador Retriever modeled using the complete Labrador Retriever dataset (860 puppies) appeared, on visual inspection, to be coherent with a steady increase in bodyweight over the observed period and smooth centiles (Fig. 2). This was confirmed when comparing the observed bodyweight distribution at each timepoint with the modeled bodyweight distribution (Fig. 3). The percentage of observed bodyweight measurements higher or lower than the values of the modeled growth centile curves was close to the target values and stable across the timepoints. The mean percentage of bodyweights lower than those for 9^th^ growth centile was 9.2% (range 6.0%–11.2%, SD 0.97, CV 10.54%), and for the 25^th^ centile this was 24.3% (range 19.5%–30.9%, SD 2.12, CV 8,73%). The mean percentage of observed bodyweights greater than those for the 75^th^ growth centile was 25.3% (range 21.4%–35.3%, SD 2.92, CV 11.56%), and for the 91^st^ centile this was 9.6% (6.1%–13.7, SD 1.47, CV 15.35%).

**Fig. 4:**
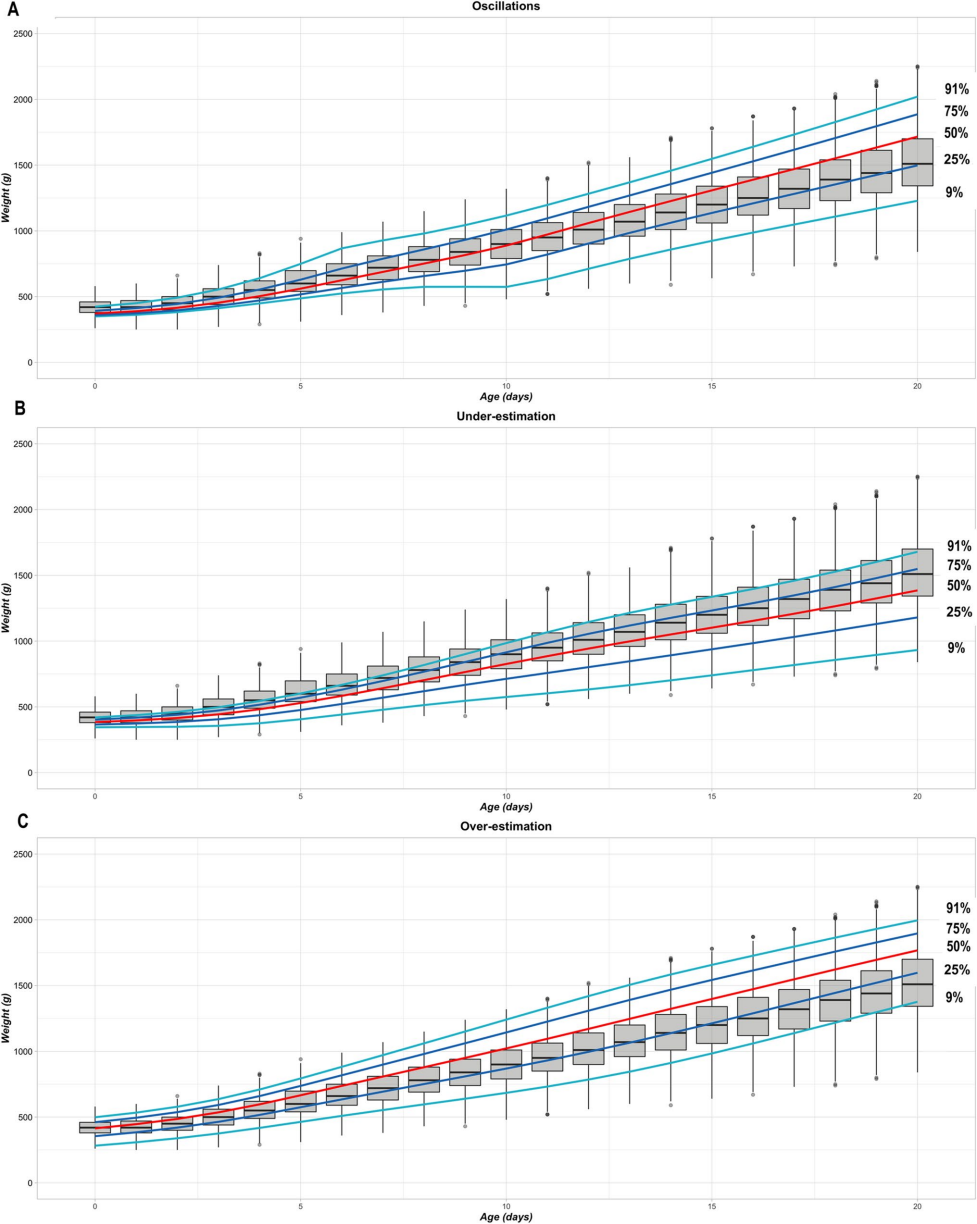
Patterns of breed-scale GCs simulated with sample sizes of three puppies. Each chart shows a different pattern of breed-scale growth centile curves for one of the 100 simulations for a sample size of three puppies from the Labrador Retriever training dataset: (**A**): oscillating pattern of growth; (**B**) underestimation of growth; (**C**) overestimation of growth. The centile curves overlay a box and whisker plot of the distribution of observed data per timepoint for the Labrador Retriever complete dataset (860 puppies). Boxes outline the interquartile range, the horizontal bars are the medians, the top and bottom whiskers extend to largest and smallest weight measures, respectively, within 1.5 times the interquartile range, and filled circles represent data points outside these ranges. BW, bodyweight; GCs, growth curves

**Fig. 5:**
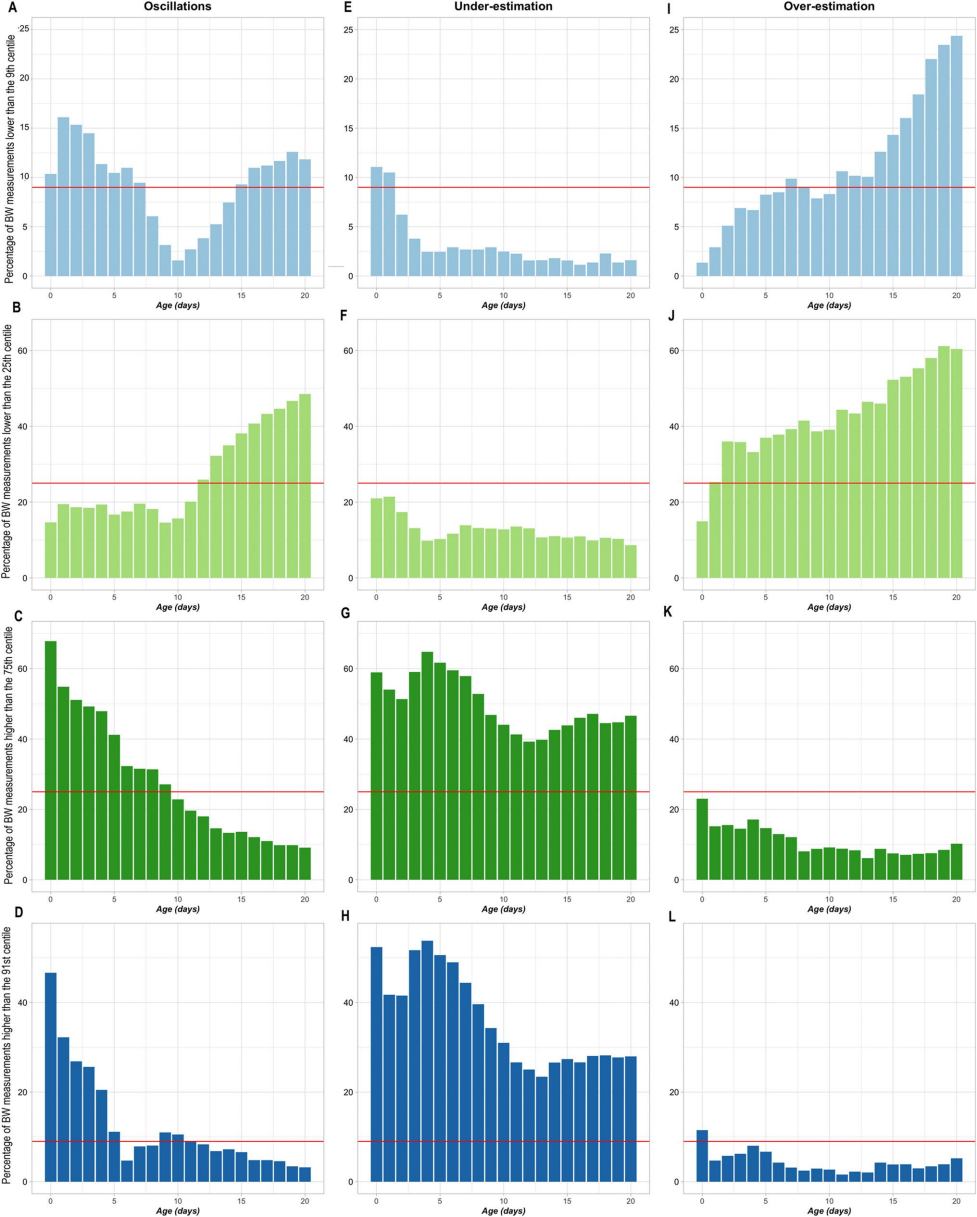
Quality estimations of breed-scale GCs simulated with sample sizes of three puppies. Charts show the percentage of the observed Labrador Retriever bodyweight measurements from the test dataset (450 puppies) by timepoint that were outside the target centiles of the breed-scale GCs modeled from sample sizes of three puppies from Labrador Retriever training datasets. Each chart series by column is an example of one of the 100 simulations representing a different pattern of quality estimations corresponding to Fig. 4. The quality estimations for the for the 9^th^, 25^th^, 75^th^, and 91^st^ centiles are given by (**A**), (**B**), (**C**), and (**D**), respectively for an oscillating pattern, by (**E**), (**F**), (**G**), and (**H**), respectively for an underestimation pattern, and by (**I**), (**J**), (**K**), and (**L**), respectively for an overestimation pattern. The red line indicates the target percentage. BW, bodyweight; GCs, growth curves

Breed-scale Labrador Retriever growth centile curves modeled from simulations of sample sizes of three puppies showed three different patterns. Typical examples of these are presented in Fig. 4; they are described as oscillating between timepoints, underestimation of growth, and overestimation of growth. These descriptions correspond to the mean percentage of observed Labrador Retriever bodyweight measurements (860 puppies) outside the growth centiles of the simulations (Fig. 5 and Online Resource 1 for corresponding statistical assessments). Variability characterizing the oscillating GCs was demonstrated by quality evaluation of the 9^th^ centile; the mean percentage of observed bodyweight measurements lower than the 9^th^ centile value ranged from 1.57% to 16.11%, resulting in a CV of 44.8% and an SD of 4.18 (Fig. 5A). A consistent underestimation of growth is demonstrated in Fig. 5E-H. Mean percentages of observed bodyweight measurements were below target for the smallest centiles (3.18% for the 9^th^ centile (Fig. 5E) and 12.71% for the 25^th^ centile (Fig. 5F)) and above target for the largest centiles (49.84% for the 75^th^ centile (Fig. 5G) and 36.08% for the 91^st^ centile (Fig. 5H)). Overestimation of growth is demonstrated in Fig. 5I-L. Mean percentages of observed bodyweight measurements were above target for the smallest centiles (11.28% for the 9^th^ centile (Fig. 5I) and 42.80% for the 25^th^ centile (Fig. 5J)) and below target for the largest (11.03% for the 75^th^ centile (Fig. 5K) and 4.38% for the 91^st^ centile (Fig. 5L)).

### Cluster-scale GCs

**Fig. 6:**
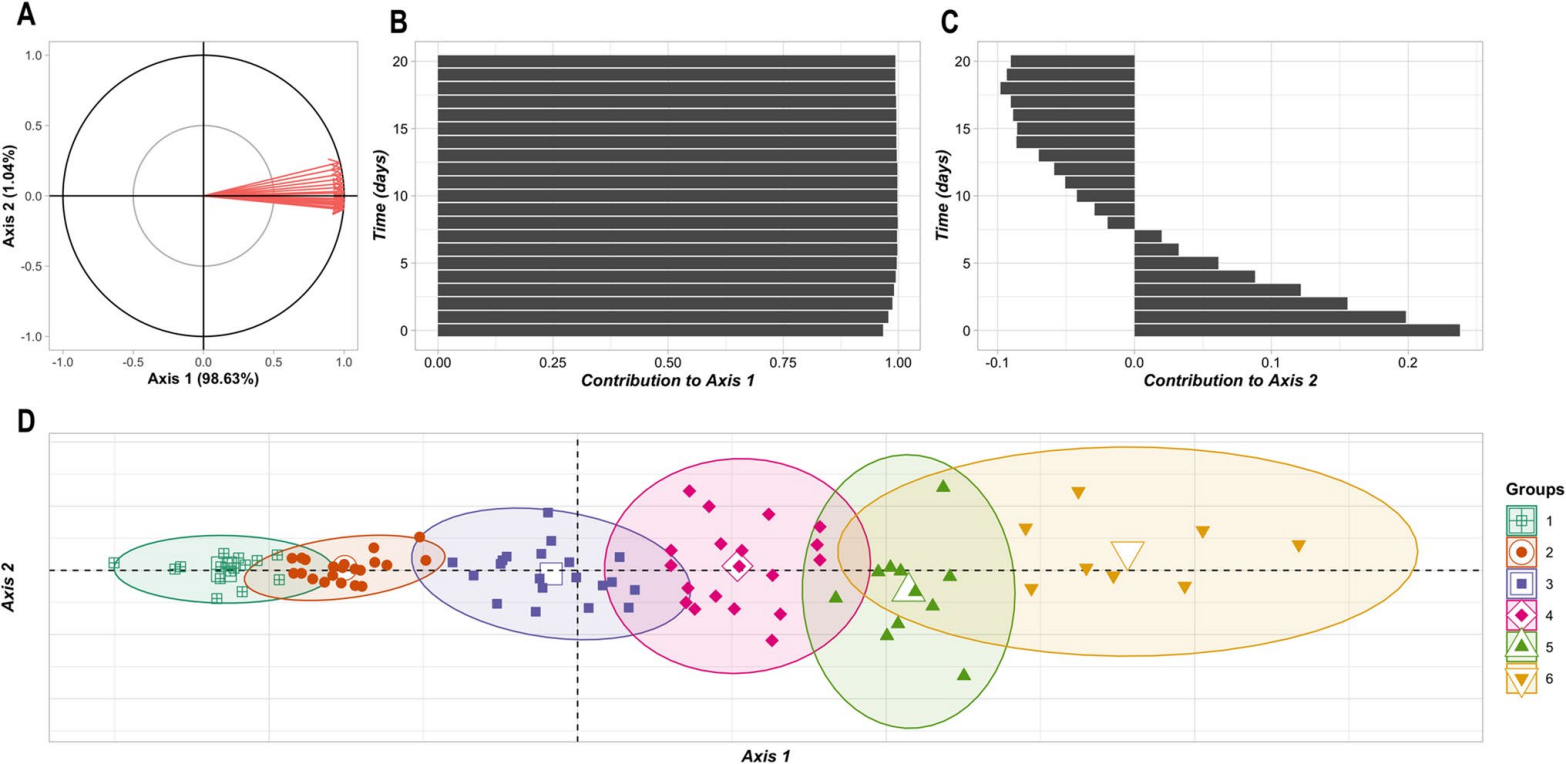
Results of unsupervised clustering of breeds. Unsupervised clustering of breeds was conducted using the global dataset of bodyweight measurements from Days 0 to 20. (**A**): the principal components analysis correlation circle. (**B**) and (**C**): the contribution of bodyweight measures to the first and second principal components, respectively. (**D**): dog breeds plotted according to the first and second principal components. Each filled shape represents a breed, and the larger unfilled shapes mark the centers of inertia (center of gravity or barycenter) of the clusters. Colors and shapes indicate the cluster to which a breed was attributed

In the unsupervised clustering of the 116 dog breeds represented in the global dataset, more than 99% of variability between breeds in bodyweight measurements was accounted for by two principal components (Fig. 6A). The first of these, which accounted for 98.63% of the variability, represented an effect of dog size, i.e., the median bodyweight of the dog breed (Fig. 6B); the second accounted for 1.04% of variability and represented the effect of puppy age (Fig. 6C). Bodyweights of puppies in the first 8 days made a positive contribution to the second principal component; after this they made a negative contribution (Fig. 6C). Clustering dog breeds using these two principal components (Fig. 6D) was equivalent to grouping breeds according to their median bodyweights and the evolution of these during the first 3 weeks.

Clustering using the global dataset defined six clusters (Fig. 6D). The breed composition of these clusters was stable when challenged by simulating clustering using reduced sample sizes for the Labrador Retriever breed. Of the total 700 clustering simulations (100 simulations for each of the seven sample sizes from the Labrador Retriever training datasets), 651 returned the same cluster containing the Labrador Retriever breed. This consisted of the following 20 breeds: Bernese Mountain Dog, Golden Retriever, German Shepherd, Boxer, White Swiss Shepherd Dog, Belgian Malinois Sheepdog, Samoyed, Dalmatian, Picardy Shepherd, Basset Hound, Akita, Dobermann, Irish Red Setter, Entlebuch Cattle Dog, Belgian Groenendael Sheepdog, Hungarian Wire-Haired Pointer, Auvergne Pointer, Continental Bulldog, French Spaniel, and Labrador Retriever.

The stability of the Labrador Retriever cluster was dependent on the Labrador Retriever sample size (Online resources 2 and 3). When Labrador Retriever sample sizes from the training datasets were 410, 200, and 100 puppies, clustering always returned the previously defined Labrador Retriever cluster. When the Labrador Retriever samples were 30, 20, and 10 puppies, this cluster composition was observed in 94% to 99% of the simulations and there was a range of 2 to 5 different Labrador Retriever clusters. With training sets of 3 puppies, this cluster was observed in 60% of the simulations and there were 15 different Labrador Retriever clusters defined across the 100 simulations.

### Comparisons between breed-scale and cluster-scale Labrador Retriever GCs with reduced sample sizes

**Fig. 7:**
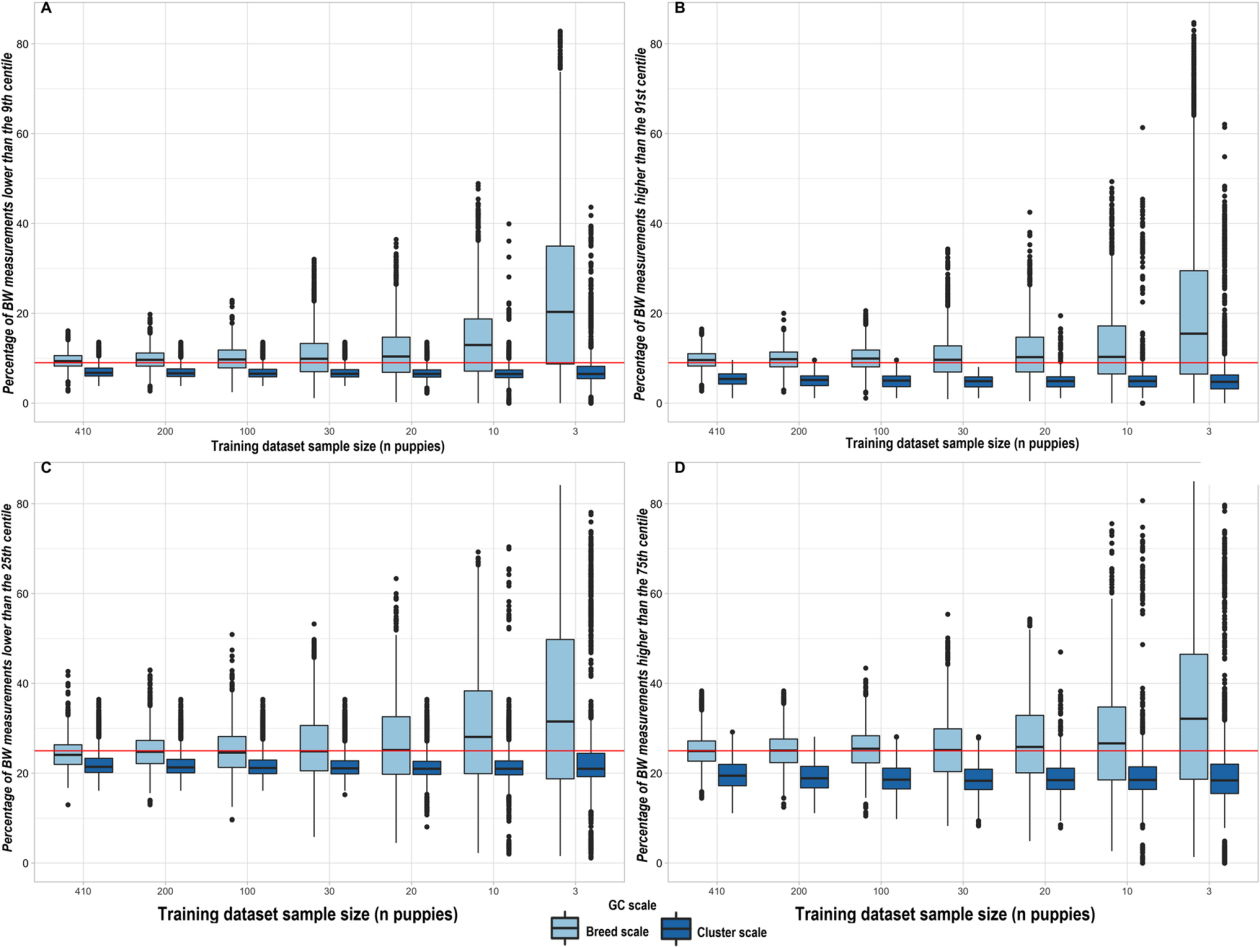
Comparison of the quality of breed-scale and cluster-scale GCs as sample sizes were reduced. Each chart shows the percentages of observed puppy bodyweights from the Labrador Retriever test dataset (450 puppies) (i.e., the quality criteria) that were outside of target quantiles for either breed- or cluster-scale breed GCs simulated with different sample sizes from the same training datasets (100 simulations for each sample size). Boxes for the quality criteria outline the interquartile ranges, the horizontal bars are the medians, the top and bottom whiskers extend to largest and smallest bodyweight measurements, respectively, within 1.5 times the interquartile ranges, and filled circles represent data points outside these ranges. The red horizonal line indicates the target percentage. The percentages of observed bodyweights shown are those that were lower than the bodyweights for the 9^th^ centile (**A**) and the 25^th^ centile (**C**), and higher than the bodyweights for the 91^st^ centile (**B**) and the 75^th^ centile (**D**). For all centiles the cluster scale had more consistent accuracy than the breed scale as sample sizes decreased, especially from sample sizes of 20 and less. GCs, growth curves

Breed-scale and cluster-scale Labrador Retriever GCs were developed with sample sizes varying from 3 to 410 puppies from the training datasets (100 simulations per sample size). Modeling quality was evaluated by comparing the percentages of bodyweight measurements in the observed test datasets (quality criteria) that were outside of target quantiles defined by the breed-scale and cluster-scale breed GCs (Fig. 7). The median quality criteria were close to all the centile targets defined by the breed-scale growth charts over the 100 simulations until the sample size was reduced to 20. Below that sample size, median quality criteria started to increase notably above the target. The interquartile distance for the quality criteria increased continuously with decreasing sample size. For cluster-scale Labrador Retriever GCs, the median quality criteria were consistently lower than the target. However, with a few exceptions, the median and interquartile distance of quality criteria were consistent until a sample size of three. These results reflected a narrowing of the breed-scale growth centile curves at low sample sizes that was much less evident for the cluster-scale GCs.

**Fig. 8:**
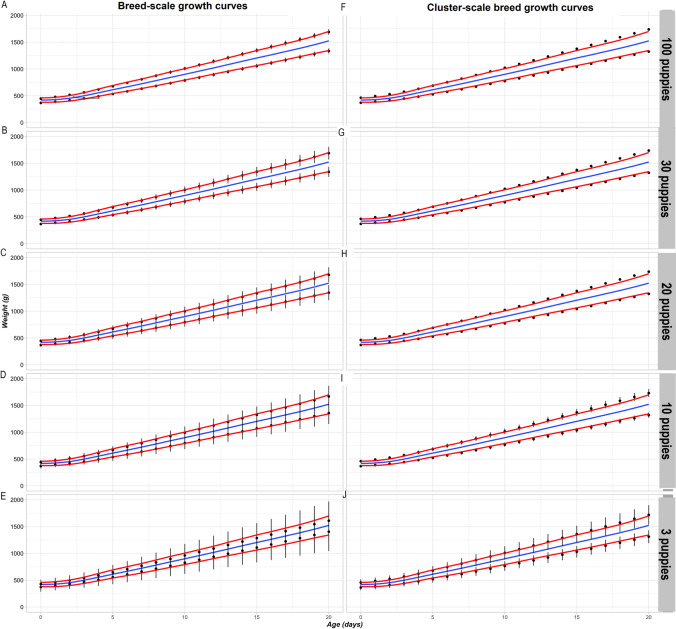
Comparisons between breed-scale and cluster-scale breed GCs. Blue lines represent the mean values per day of observed bodyweight measurements in the complete Labrador Retriever dataset (860 puppies). Red lines are the observed 25^th^ and 75^th^ centiles for the same dataset. Black points are the mean values per timepoint of the 25^th^ and 75^th^ growth curve centiles obtained from 100 modeling simulations for each sample size; black error bars correspond to the 95% confidence intervals (2 times the standard deviation). The chart series **A**, **B**, **C**, **D** and **E** shows data for breed-scale GCs modeled from sample sizes of 100, 30, 20, 10 and 3 puppies, respectively; the chart series **F**, **G**, **H**, **I** and **J** shows the equivalent data for cluster-scale breed GCs. The cluster scale shows a slightly greater spread of centiles than for observed data but these are stable with decreasing sample size. The breed scale shows a notable narrowing of centile curves at a sample size of three. GCs, growth curves

Breed-scale and cluster-scale Labrador Retriever GCs modeled with decreasing sample sizes were also compared by plotting mean (95% CI) bodyweight measurements for the 25^th^ and 75^th^ centiles across 100 simulations for each scale and sample size on charts that also showed the mean, and 25^th^ and 75^th^ centile values of observed bodyweight measurements in the complete Labrador Retriever dataset (860 puppies) (Fig. 8). As sample sizes for modeling simulations decreased, the distance between the mean 25^th^ and 75^th^ centiles of the breed-scale GCs narrowed compared with the distance between the 25^th^ and 75^th^ centiles of the observed bodyweight measurements; associated 95% CIs increased (Fig. 8A-E). In contrast, the distance between mean 25^th^ and 75^th^ centiles obtained from cluster-scale breed growth charts was slightly greater than the observed 25^th^ and 75^th^ centiles but remained stable when sample size progressively decreased; associated 95% CIs remained relatively small compared with those of the breed-scale GCs.

## Discussion

This study introduces a methodological approach to building puppy growth centile curves that satisfies the requirement for breed specificity and overcomes the limitations of small sample sizes for some breeds. Previous studies have addressed these challenges by developing GCs based on breed sizes defined by adult bodyweight (Hawthorne et al. 2004; Salt et al. 2017). This allowed no consideration of breed-specific patterns of development. Nevertheless, in a study modeling GCs for puppies from the age of 2 months, a good agreement was found between size-scale GCs and breed-scale GCs for those breeds that had sufficient data to model the latter (Salt et al. 2017). This suggests that post-weaning growth of puppies can be satisfactorily described using size categories. That study initially developed models using five previously proposed adult-size categories (extra-small, mini, medium, maxi and giant) (Hawthorne et al. 2004). However, the model for one of the size groups had a bimodal residual distribution, and the largest breeds in two of the other size groups had an excessive influence on the models (Salt et al. 2017). These anomalies were resolved by refining breed groupings into six new size classes (I to VI) using cluster analysis of the growth data in a similar way to the clustering reported here. Our study complements this previous work by Salt et al. (Salt et al. 2017) by addressing the growth of puppies prior to 2 months of age, when it is believed that breed-specific development cannot be generalized meaningfully at the level of adult size categories. The physiology of the neonate is unique (Grundy 2006), and differences in bodyweight growth patterns between breeds within size clusters have been reported in young puppies (Hawthorne et al. 2004). Our study is also different because data for puppies prior to weaning came from individual breeders; the global dataset of approximately 16,000 puppies was therefore much smaller than that used by Salt et al., who were able to leverage large datasets of more than 44,000 puppies from veterinary clinics.

Modeling of the complete Labrador Retriever dataset of bodyweight measurements from birth to Day 20 (n = 860 puppies) produced relevant breed-scale growth centile curves (Fig. 2). The accuracy was confirmed quantitatively by determining the percentage of observed bodyweights that met the 9^th^, 25^th^, 75^th^, and 91^st^ centile ‘targets’ of the modeled GCs (Fig. 3). However, small sample sizes for a breed could result in poor modeling quality at the breed scale. This manifested visually as oscillating, underestimated or overestimated growth centile curves for different individual simulations (Fig. 4), and the unsatisfactory quality of these curves was quantified using the modeling quality criteria from the full observed dataset (Fig. 5).

These problems of small sample size for any single breed led us to investigate a hybrid methodology for growth curve modeling that used a combination of breed-specific data and data from breeds grouped by statistical unsupervised clustering rather than adult size, previously shown to produce more accurate models (Salt et al. 2017). The key results of this study demonstrate that cluster-scale breed GCs can be generated with small sample sizes and remain representative of the breed population.

Quality estimations for breed-scale growth curve simulations showed that GAMLSS modeling was not satisfactory with sample sizes of 20 or fewer per timepoint, with respect to both increasing variability in the criteria and increasing divergence of their median values from the target (Fig. 7). This was also evident from visual inspection of simulated breed-scale growth centile curves for small sample sizes, which became closer together the smaller the sample size (Fig. 8). These growth curve characteristics indicate an underestimation of population variability and more globally a misestimation of the breed population bodyweight distribution.

In contrast, simulations of cluster-scale breed GCs demonstrated that the cluster methodology was satisfactory with breed-specific sample sizes as small as three puppies. Compared with breed-scale GCs for small sample sizes, the quality criteria were closer to the targets, were similar across sample sizes and had symmetric inter-quantile distances for opposite quantiles. Clusters comprised breeds with similar median bodyweight growth profiles but potentially different bodyweights and adult sizes. Centering breed-specific data in a cluster to the cluster median profile meant that cluster-scale breed GCs could be modeled with the benefit of the combined data from all breeds in the cluster while accounting for differences in median bodyweights between breeds. Recentering the cluster GCs back to each median breed profile reinstated breed bodyweight differences, which would reduce the inter-quantile distances and make cluster-scale breed GCs a better approximation of breed-scale GCs.

The shift in quality criteria for the cluster-scale Labrador Retriever growth centiles to values slightly lower than the target (Fig. 7) was attributed to a moderate increase in variability introduced from other breeds within the cluster. Stable values for the median quality criteria and the variability of quality estimations show that the cluster-scale breed GCs were much less impacted by sample size than breed-scale GCs were. It suggests that modeling quality of cluster-scale breed GCs was driven more by the cluster sample than by the breed sample, regardless of the breed sample size. Consequently, when validating the clustering methodology, attention must be given to the cluster size and composition (repartition across timepoints) as well as to the cluster-scale GCs themselves.

Cluster definition was not strongly impacted by sample size. This stability can be attributed to the fact that the statistical unit of the clustering method was the breed median growth profile, which is independent of the sample size of bodyweight measurements for the breed. Nevertheless, greater variability in the cluster definition was observed with the smallest sample size (three puppies). Indeed, with such a sample size, the median growth profile could not be representative of the breed population growth profile. Differences between sample sizes of the breeds comprising a cluster could also lead to an under- or over-representation of breeds and thereby bias the modeled GCs. Further development of the methodology could introduce a sampling method to balance sample size of the different breed.

This study used Labrador Retriever training and test datasets comprising only puppies that had all daily bodyweight measures from birth to Day 20. Restricting modeling simulations to this period increased the maximum sample size and facilitated the generation of subsets by sampling puppies. The study did not investigate whether the accuracy of GCs was impacted by sample composition in terms of distribution of bodyweight measurements across time points. Sample composition could also impact the breed median growth profile and consequently the cluster definition as well as the centering of cluster GCs to the breed median growth profile. These limitations of the methodology may be managed by controlling the homogeneity of the sample composition, and by graphically validating the generated growth centile curves. The impact of sample composition on the quality of growth centile curves should be quantified in further studies.

## Conclusions

These results demonstrate with the example of the Labrador Retriever that clustering breeds on the basis of median growth profiles is a potentially valuable approach to modeling GCs when there are insufficient data to model good quality breed-scale GCs by GAMLSS. The accuracy of GCs at breed scale became unsatisfactory when the sample size was 20 puppies or less per time point. In contrast, the quality of cluster-scale breed GCs was robust with sample sizes of only three bodyweight measurements per time point. Subject to further validation, the option of using this methodology could enable the availability of breed-representative GCs for all canine breeds prior to weaning, including breeds that are numerically poorly represented.

## Supplementary Information

Below is the link to the electronic supplementary material.

Supplementary file1 (PDF 50 KB)

Supplementary file2 (PDF 62 KB)

Supplementary file3 (PDF 56 KB)

## Data Availability

The datasets generated during and/or analyzed during the current study are available from the corresponding author on reasonable request.
