# Supplementary material for: Growth charts for small sample sizes using unsupervised clustering: Application to canine early growth
Source: Vet Res Commun. 2022 Nov 5;47(2):693–706. doi: 10.1007/s11259-022-10029-2 (PMC10209281; doi:10.1007/s11259-022-10029-2)
Supplement: Supplementary file 1 — Supplementary file1 (PDF 50 KB) [file 11259_2022_10029_MOESM1_ESM.pdf]

On-line Supplement 1

Growth charts for small sample sizes using unsupervised clustering: Application to canine early growth

Gabriel Kocevar[1], Maxime Rioland[1], Jérémy Laxalde[2], Amélie Mugnier[3], Achraf Adib-Lesaux[2], Virginie Gaillard[2 *], Jonathan Bodin[1]

[1] Seenovate, Lyon, France

[2] Royal Canin Research Center, Aimargues, France

[3] NeoCare, Université de Toulouse, ENVT, Toulouse, France

*Corresponding author. Email: virginie.gaillard@royalcanin.com

Journal: Veterinary Research Communications

Quality estimations for examples of different patterns of breed-scale growth curves simulated with a sample size of three puppies

Example simulated GC pattern	Percentage of observed BW measurements lower or higher than the target centile of a breed-scale Labrador Retriever growth curve simulation Mean % (±SD) [CV%]			
	Lower than 9th centile	Lower than 25th centile	Higher than 75th centile	Higher than 91st centile
**Over-estimation**	11.28 (±6.38) [56.59%]	42.80 (±11.47) [26.80%]	11.03 (±4.29) [38.86%]	4.38 (±2.31) [52.86%]
**Under-estimation**	3.18 (±2.75) [86.68%]	12.71 (±3.42) [26.90%]	49.84 (±7.81) [15.66%]	36.08 (±10.63) [29.46%]
**Oscillating**	9.34 (±4.18) [44.78%]	27.04 (±12.04) [44.51%]	28.02 (±17.68) [63.13%]	12.57 (±11.36) [90.35%]

BW, bodyweight; CV, coefficient of variation; GC, growth curve; SD, standard deviation.
