# Supplementary material for: Growth charts for small sample sizes using unsupervised clustering: Application to canine early growth
Source: Vet Res Commun. 2022 Nov 5;47(2):693–706. doi: 10.1007/s11259-022-10029-2 (PMC10209281; doi:10.1007/s11259-022-10029-2)
Supplement: Supplementary file 2 — Supplementary file2 (PDF 62 KB) [file 11259_2022_10029_MOESM2_ESM.pdf]

On-line Supplement 2

Growth charts for small sample sizes using unsupervised clustering: Application to canine early growth

Gabriel Kocevar[1], Maxime Rioland[1], Jérémy Laxalde[2], Amélie Mugnier[3], Achraf Adib-Lesaux[2], Virginie Gaillard[2 *], Jonathan Bodin[1]

[1] Seenovate, Lyon, France

[2] Royal Canin Research Center, Aimargues, France

[3] NeoCare, Université de Toulouse, ENVT, Toulouse, France

*Corresponding author. Email: virginie.gaillard@royalcanin.com

Journal: Veterinary Research Communications

Labrador Retriever clusters derived from unsupervised clustering simulations for each Labrador Retriever sample size

Cluster	Sample size of Labrador Retrievers from the training datasets						
	410	200	100	30	20	10	3
1	100	100	100	98	99	94	60
2	0	0	0	2	1	1	6
3	0	0	0	0	0	3	5
4	0	0	0	0	0	1	1
5	0	0	0	0	0	1	6
6	0	0	0	0	0	0	2
7	0	0	0	0	0	0	7
8	0	0	0	0	0	0	3
9	0	0	0	0	0	0	2
10	0	0	0	0	0	0	2
11	0	0	0	0	0	0	1
12	0	0	0	0	0	0	1
13	0	0	0	0	0	0	2
14	0	0	0	0	0	0	1
15	0	0	0	0	0	0	1

There were 100 simulations for each sample size for Labrador Retrievers. All data from puppies of other breeds were included in the clustering (Days 0 to 20).
