# Supplementary material for: Growth charts for small sample sizes using unsupervised clustering: Application to canine early growth
Source: Vet Res Commun. 2022 Nov 5;47(2):693–706. doi: 10.1007/s11259-022-10029-2 (PMC10209281; doi:10.1007/s11259-022-10029-2)
Supplement: Supplementary file 3 — Supplementary file3 (PDF 56 KB) [file 11259_2022_10029_MOESM3_ESM.pdf]

On-line Supplement 3

Growth charts for small sample sizes using unsupervised clustering: Application to canine early growth

Gabriel Kocevar[1], Maxime Rioland[1], Jérémy Laxalde[2], Amélie Mugnier[3], Achraf Adib-Lesaux[2], Virginie Gaillard[2 *], Jonathan Bodin[1]

[1] Seenovate, Lyon, France

[2] Royal Canin Research Center, Aimargues, France

[3] NeoCare, Université de Toulouse, ENVT, Toulouse, France

*Corresponding author. Email: virginie.gaillard@royalcanin.com

Journal: Veterinary Research Communications

Breed composition of Labrador Retriever clusters defined by unsupervised clustering simulations for each Labrador Retriever sample size

**Cluster 1**	Bernese Mountain Dog, Golden Retriever, German Shepherd, Boxer, White Swiss Shepherd Dog, Belgian Malinois Sheepdog, Samoyed, Dalmatian, Picardy Shepherd, Basset Hound, Akita, Dobermann, Irish Red Setter, Entlebuch Cattle Dog, Belgian Groenendael Sheepdog, Hungarian Wire Haired Pointer, Auvergne Pointer, Continental Bulldog, French Spaniel, Labrador Retriever
**Cluster 2**	Bernese Mountain Dog, Australian Shepherd, Polish Lowland Sheepdog, Golden Retriever, German Shepherd, Boxer, Australian Cattle Dog, White Swiss Shepherd Dog, Belgian Malinois Sheepdog, Samoyed, Dalmatian, Picardy Shepherd, Eurasian, Basset Hound, Akita, Dobermann, Collie Smooth, Irish Red Setter, Entlebuch Cattle Dog, Belgian Groenendael Sheepdog, Hungarian Wire Haired Pointer, Auvergne Pointer, Continental Bulldog, French Spaniel, Labrador Retriever
**Cluster 3**	Australian Shepherd, Welsh Corgi Pembroke, Beagle, Polish Lowland Sheepdog, Kerry Blue Terrier, Golden Retriever, Bulldog, Poodle Royal, Boston Terrier, Long Haired Pyrenean Sheepdog, Border Collie, Australian Cattle Dog, Whippet, Samoyed, Bearded Collie, Eurasian, Shiba, Rottweiler, Bull Terrier, Schnauzer, Collie Smooth, Collie Rough, Small Munsterlander Pointer, English Setter, Labrador Retriever
**Cluster 4**	Australian Shepherd, Polish Lowland Sheepdog, Australian Cattle Dog, Eurasian, Shiba, Bull Terrier, Collie Smooth, English Setter, Labrador Retriever
**Cluster 5**	Newfoundland, Leonberg, Beauce Sheep Dog, Briard, Shar Pei, Siberian Husky, Saarloos Wolfhond, Giant Schnauzer, Greyhound, Great Swiss Mountain Dog, Labrador Retriever
**Cluster 6**	Bernese Mountain Dog, Newfoundland, German Shepherd, Leonberg, Beauce Sheep Dog, Briard, Shar Pei, Siberian Husky, Entlebuch Cattle Dog, Saarloos Wolfhond, Hungarian Wire Haired Pointer, Giant Schnauzer, Greyhound, Great Swiss Mountain Dog, Labrador Retriever
**Cluster 7**	Australian Shepherd, Welsh Corgi Pembroke, Beagle, Polish Lowland Sheepdog, Kerry Blue Terrier, Bulldog, Poodle Royal, Boston Terrier, Long Haired Pyrenean Sheepdog, Border Collie, Australian Cattle Dog, Whippet, Bearded Collie, Eurasian, Shiba, Rottweiler, Bull Terrier, Schnauzer, Collie Smooth, Collie Rough, Small Munsterlander Pointer, English Setter, Labrador Retriever

**Cluster 8**	Newfoundland, Leonberg, Beauce Sheep Dog, Briard, Shar Pei, Siberian Husky, Saarloos Wolfhond, Hungarian Wire Haired Pointer, Giant Schnauzer, Greyhound, Great Swiss Mountain Dog, Labrador Retriever
**Cluster 9**	Bernese Mountain Dog, Golden Retriever, German Shepherd, Boxer, White Swiss Shepherd Dog, Belgian Malinois Sheepdog, Samoyed, Dalmatian, Picardy Shepherd, Basset Hound, Akita, Dobermann, Irish Red Setter, Entlebuch Cattle Dog, Belgian Groenendael Sheepdog, Auvergne Pointer, Continental Bulldog, French Spaniel, Labrador Retriever
**Cluster 10**	Australian Shepherd, Beagle, Polish Lowland Sheepdog, Bulldog, Poodle Royal, Boston Terrier, Long Haired Pyrenean Sheepdog, Border Collie, Australian Cattle Dog, Bearded Collie, Eurasian, Shiba, Rottweiler, Bull Terrier, Collie Smooth, Collie Rough, Small Munsterlander Pointer, English Setter, Labrador Retriever
**Cluster 11**	Australian Shepherd, Polish Lowland Sheepdog, Golden Retriever, Boxer, Australian Cattle Dog, White Swiss Shepherd Dog, Belgian Malinois Sheepdog, Samoyed, Dalmatian, Eurasian, Basset Hound, Dobermann, Collie Smooth, Belgian Groenendael Sheepdog, Auvergne Pointer, Continental Bulldog, French Spaniel, Labrador Retriever
**Cluster 12**	Australian Shepherd, Polish Lowland Sheepdog, Golden Retriever, Boxer, Australian Cattle Dog, White Swiss Shepherd Dog, Belgian Malinois Sheepdog, Samoyed, Dalmatian, Eurasian, Basset Hound, Shiba, Dobermann, Collie Smooth, Belgian Groenendael Sheepdog, Auvergne Pointer, Continental Bulldog, French Spaniel, Labrador Retriever
**Cluster 13**	Golden Retriever, Boxer, White Swiss Shepherd Dog, Belgian Malinois Sheepdog, Samoyed, Dalmatian, Eurasian, Basset Hound, Dobermann, Collie Smooth, Belgian Groenendael Sheepdog, Auvergne Pointer, Continental Bulldog, French Spaniel, Labrador Retriever
**Cluster 14**	Boxer, White Swiss Shepherd Dog, Basset Hound, Dobermann, Auvergne Pointer, Labrador Retriever
**Cluster 15**	Bullmastiff, Dogo Argentino, Dogue De Bordeaux, Labrador Retriever
